# Behavioural responses of *Anopheles gambiae* to standard pyrethroid and PBO-treated bednets of different operational ages

**DOI:** 10.1016/j.crpvbd.2024.100227

**Published:** 2024-11-04

**Authors:** Emma Reid, Frank Mechan, Jeff Jones, Amy Lynd, Janet Hemingway, Philip McCall, David Weetman

**Affiliations:** Liverpool School of Tropical Medicine, Pembroke Place, Liverpool, L3 5QA, UK

**Keywords:** *Anopheles gambiae*, Insecticide, Behaviour, Piperonyl butoxide (PBO), Insecticide-treated nets (ITN)

## Abstract

To combat pyrethroid insecticide resistance, there has been widespread distribution of pyrethroid-treated bednets (ITNs) co-impregnated with piperonyl butoxide (PBO), a synergist that inhibits enzyme activity to block metabolic resistance. While PBO impacts physiological resistance, mosquito behavioural responses when attempting to blood-feed through nets may be more dependent on net characteristics, in particular the insecticide treatment and operational age of nets. These potentially interacting effects are currently not well characterised. This study aimed to investigate the behavioural responses of *Anopheles gambiae* to different types of ITNs of different ages to evaluate the relationships between behaviours, insecticide type, age of net and mortality. A pyrethroid-resistant *An. gambiae* strain originally from Busia, Uganda, was tested with modified WHO cone assays in which a human arm is provided as bait and the trial is video recorded. Using the recordings, movement patterns throughout the cone were monitored to assess net contact and avoidance behaviours. Nets tested were PermaNet 2.0, PermaNet 3.0, Olyset and Olyset Plus, aged 0 months (unused), 12 months, and 25 months post-deployment, all collected from a field trial in Uganda. Our primary hypothesis was that behavioural indices of irritancy would decline with net age as active ingredient concentrations decline, in line with mortality. Knockdown and mortality were highest on baseline nets with PBO and declined thereafter, whereas each was much lower and invariant with age for non-PBO nets. Mosquito movement in the cones was also higher at baseline and declined with age for PBO nets, but not non-PBO nets, indicating an association between mortality and irritancy-induced movement. Baseline nets with PBO also elicited less net contact than older nets, whilst non-PBO nets showed no relationship between net contact and age. PBO nets also elicited irritancy behaviour even after a short period of exposure. In conclusion, the addition of PBO was initially effective in restoring the efficacy of nets, but this relative advantage declined with time, as did the behavioural indices, movement and net contact, suggesting declining irritancy as PBO is lost.

## Highlights

• Mosquitoes made less contact to PBO nets than standard pyrethroid nets.
• More mosquito movement was detected with PBO nets, which declined with net age.
• Nets with higher mosquito movement had more knockdown and mortality.

## Graphical abstract

**Figure:**
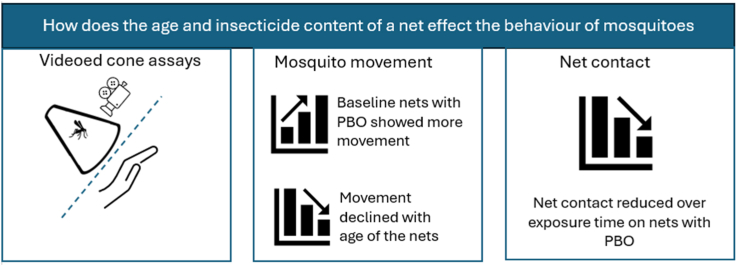

## 1 Introduction

The use of insecticidal bednets has been a key factor in reducing the malaria burden in Africa (Bhatt et al., 2015). Insecticide-treated nets (ITNs) work by protecting individuals with a physical barrier against blood-seeking female mosquitoes, reducing mosquitoes’ contact with the net *via* irritation upon contact caused by the insecticide (sometimes termed “excito-repellency”), and killing the mosquitoes when they encounter the net (Parker et al., 2015). Pyrethroids are currently used in ITNs because of their favourable human safety profile, rapid knockdown, and killing (Aizoun et al., 2013). ITNs are intended to provide protection for three years and withstand repeated washing (Sousa et al., 2019).

All bed nets are treated with either a type I (permethrin) or type II (deltamethrin, alphacypermethrin) pyrethroid; the latter which include an alpha-cyano moiety intended to increase potency. Owing to widespread and rising levels of resistance (Ranson and Lissenden, 2016; WHO, 2018) to all pyrethroids, second-generation nets have been produced which include either a combination of pyrethroid with a second insecticide or piperonyl butoxide (PBO) as an interim whilst new insecticides are developed (WHO, 2019). The addition of PBO is intended to improve the effectiveness of the net by inhibiting metabolic enzymes (particularly P450s) within mosquitoes, which detoxify insecticides and can cause pyrethroid resistance (Ngufor et al., 2022). Following successful trials in northeastern Tanzania (Protopopoff et al., 2018) and Uganda (Staedke et al., 2020) WHO recommended considering deployment of PBO nets in areas of high pyrethroid resistance, and deployment of PBO nets in sub-Saharan Africa increased to 46% in 2021 (Ngufor et al., 2022; WHO, 2022; Gleave et al., 2023). However, there are concerns about the insecticide lifespan of PBO nets in the field, with recent trial evidence suggesting a relative decline in performance after one year of operational use (Mosha et al., 2022). Loss of PBO from nets is likely a major contributory factor, with PBO declining more rapidly than permethrin in co-treated Olyset plus nets (Gichuki et al., 2021; Lukole et al., 2022; Mechan et al., 2022) or deltamethrin (Mechan et al., 2022) in PermaNet 3.0 nets. This is associated with declines in mosquito mortality in standard WHO cone tests (Mechan et al., 2022). Owing to their wide distribution in malaria control programmes in sub-Saharan Africa, a better understanding of the factors which may lead to performance changes over time are needed.

Although physiological insecticide resistance is a well-recognised problem, behavioural avoidance may also limit the efficacy of ITNs by allowing vectors to reduce insecticide update by minimising contact time with toxic surfaces (Kreppel et al., 2020). There are thought to be two broad categories of behavioural avoidance either by irritancy elicited by direct contact, where the target vector leaves after contact, or by non-contact spatial repellency, where avoidance occurs without contact with the treated surface (Roberts et al., 1997; Kreppel et al., 2020); however, which of these may be induced by bednets is not well established. Room-scale video tracking has provided an assessment of the behaviour of host-seeking *Anopheles* mosquitoes around different types of bednets, showing reductions in the number and duration of net contacts and declining activity of susceptible strains exposed to treated (standard and PBO- or dual-treated) *vs* untreated nets (Gleave et al., 2023). Whilst such in-depth investigations provide detailed assessments of behavioural modifications of mosquitoes exposed to bednets, specialised equipment is required and throughput is low. Consequently, ‘bench-top’ behavioural assays have been developed to provide tractable, higher-throughput methodologies to study behavioural variation in proximity to ITNs (Hughes et al., 2020, 2022; Jones et al., 2023). These assays offer the advantage of providing a human host stimulus to elicit realistic responses compared to the standard WHO cone assays.

It is important to understand this decline in net performance over the distribution cycle, as well as the potentially interacting effect of net type and operational age on mosquito behaviour during host-seeking through a net. Here we investigate how behaviour and mortality varied with ITN post-deployment age and type using video cone assays, a modification of the standard WHO cone test, which uses a human arm as bait and records mosquito behaviour (Hughes et al., 2022; Jones et al., 2023).

The nets tested in this study, came from a randomised control trial in Uganda (Staedke et al., 2019), from which nets were withdrawn at baseline, 12 months, and 25 months. Four types of nets, two pyrethroid-only and two pyrethroid + PBO ITNs, that had previously undergone WHO cone assay tests (Mechan et al., 2022), were analysed using the video cone method to assess behavioural differences of resistant *Anopheles gambiae*, originating from Uganda, to different ITNs of varying post-deployment age and type.

## 2 Materials and methods

### 2.1 Mosquitoes

The mosquitoes used in this study were *Anopheles gambiae* (*s.s.*), originating from Busia, Uganda. This strain was established from field collections in November 2018 and has been maintained at the Liverpool School of Tropical Medicine since. Mosquitoes were reared in environmental controlled insectaries at temperatures of 25–27 °C (min-max) and a relative humidity of 70–80%. The strain is resistant to pyrethroids, with alterations in the target site (*Vgsc-*1014S) and metabolic resistance mechanisms (*Cyp4j5*, *Cyp6aa1*, and *Coea1d*) (Weetman et al., 2018; Njoroge et al., 2021; Mechan et al., 2022). Selections were performed every five generations, using 0.05% deltamethrin to maintain resistance. Unfed female mosquitoes aged 3–5 days were used for testing. Approximately 20 h prior to testing, mosquitoes were starved by replacing cotton sugar pads with water, which were removed 4 h prior to testing to increase host-seeking behaviour. After the tests, the sugar pads were replaced during the recovery period. At the start and end of each test day, negative control cones were performed with untreated netting using similarly starved mosquitoes all of which had 0% mortality.

### 2.2 ITNs evaluated

The ITNs tested in this study were obtained from a cluster-randomised trial in Uganda (Staedke et al., 2020). Four ITNs from the Uganda campaign were used: PermaNet 2.0, PermaNet 3.0, Olyset, and Olyset Plus. All the samples were collected from the top portion of the nets. Nets were collected from the field at baseline, 12 months, and 25 months post-deployment which hereafter will be referred to as age. The methods for deployment of nets during the campaign have been previously discussed by Staedke et al. (2019). Full description of the treatment on the nets and material is provided in Table 1.

**Table 1:** Description of the net samples used.

Net type	Manufacturer	Treatment	Denier	Material
PermaNet 2.0	Vestergaard Sarl	1.4 ± 0.35 g/kg of deltamethrin	100	Polyester
PermaNet 3.0	Vestergaard Sarl	4.0 ± 1.0 g/kg deltamethrin and 25 ± 2.5 g/kg PBO	100	Polyester
Olyset Net	Sumitomo Chemical Ltd	20 ± 5.0 g/kg permethrin	150	Polyethylene
Olyset Plus	Sumitomo Chemical Ltd	20 ± 5.0 g/kg permethrin and 10 ± 2.5 g/kg PBO	150	Polyethylene

### 2.3 Video cone bioassay

**Fig. 1:**
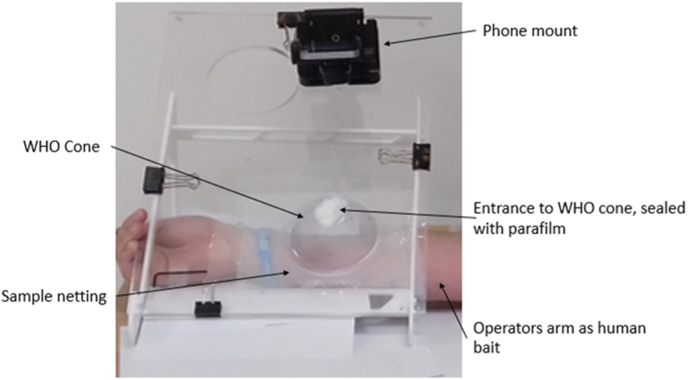
Video cone assay equipment setup displaying a phone mounted on boards with a bait arm underneath.

This assay is an adapted version of the WHO cone bioassay (WHO, 2013). Mosquito exposures were conducted using the apparatus shown in Fig. 1, which comprises a frame holding a plastic board at a 45° angle (Owusu and Muller, 2016) with enough room to allow for an arm to rest underneath as bait throughout the test. Each net sample was attached to the board over a 9 cm circular hole and a standard WHO cone with a base diameter of 12 cm (WHO, 2023) placed on top. Mosquitoes were inserted using an aspirator, into the cone from the entrance, as shown in Fig. 1 and sealed using parafilm. A mobile phone (in this case, an iPhone 7) is clamped into the phone mount, and using the video camera function, the test is recorded for a 3 min exposure time, which is the standard for WHO cone tests (WHO, 2011), the recording was manually started and stopped by the operator. Mosquitoes were blown using an aspirator into cups after exposure, and 1-h knockdown and 24-h mortality were recorded. All the tests were conducted at 27 ± 5 °C and 80 ± 5%) relative humidity. Each test used five mosquitoes and was replicated ten times.

### 2.4 Data collection and analysis

Video files collected were analysed using two different methods, scan sampling method with the Behavioural Observation Research Interactive Software (BORIS) developed by the University of Torino (Friard and Gamba, 2016) and Video Cone Test Analysis (ViCTA) software developed at the Liverpool School of Tropical Medicine (Jones et al., 2023). After being recorded, the videos were converted to 240 × 590 pixels with 30 frames per second.

For the scan sampling method (Hughes et al., 2022) the video was stopped every 5 s and the numbers of mosquitoes on the net and elsewhere in the cone were counted using BORIS software. The data were then exported to Excel.

The ViCTA software uses background subtraction methods to detect moving mosquitoes in the video footage at 0.1-s intervals and aggregates these counts to 5-s intervals to match the scan-sampling intervals. The data points are referred to as movement events. A comparison of the two methods is shown in Table 2.

**Table 2:** Comparison of the features of the data collection methods.

Feature	Scan-sampling	ViCTA
Analysis method	Subjective (manual)	Objective (automated)
Data type	Behavioural state (on the net, not on the net)	Detected mosquito movement
Data points	37 (1 every 5 s)	1800 (1 every 0.1 s) aggregated to 5-s intervals
Time taken for video analysis	10–20 min per replicate	30 s to 2 min per replicate (depending on PC)

### 2.5 Statistical analysis

Data analysis was conducted using R (version 4.4.1), with the *ggplot2* package (version 3.5.1) was used to produce all graphs. Generalised linear mixed-effects models (GLMMs) were used to quantify associations between outcomes and variables of interest with replicates included in the models as a random effect (using the *lme4* package version 1.1.35.5).

## 3 Results

### 3.1 Knockdown and mortality

#### 3.1.1 With a host

**Fig. 2:**
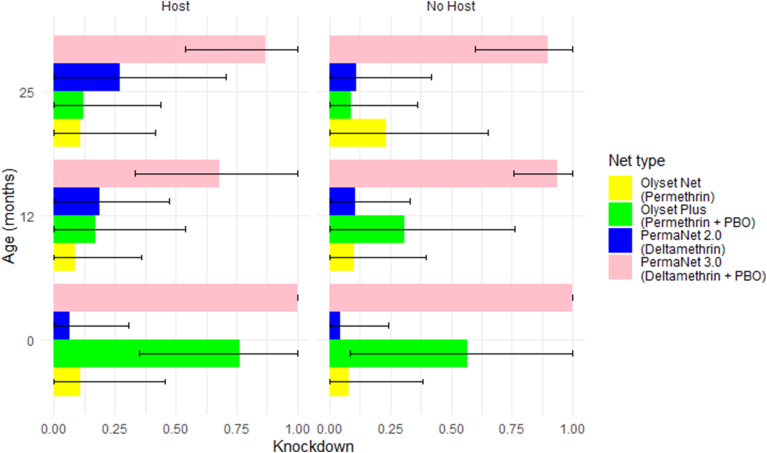
Bar charts displaying the proportion knockdown with and without a host for the four net types with 95% confidence intervals determined by the best-fit model.

**Fig. 3:**
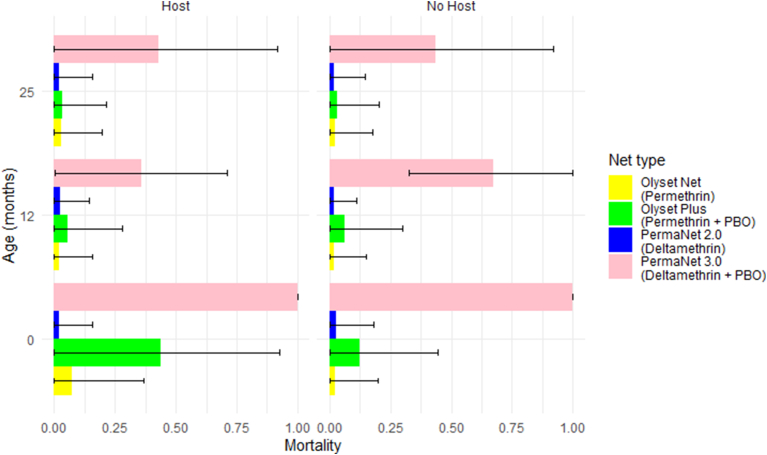
Bar plots displaying proportion mortality for all four net types with and without a host, with 95% confidence intervals determined by the best-fit model.

The PBO nets were most effective at baseline but declined with operational use. Knockdown for PermaNet 3.0 was 100% at baseline, 68% (95% CI: 33.44–100%) at 12 months and 87% (95% CI: 54.04–100%) at 25 months (Fig. 2). However, no significant differences were found between baseline and 12 months (*P* = 0.42), baseline and 24 months (*P* = 1.00) or 12 and 25 months (*P* = 0.65). A stronger reduction was observed for 24-h mortality; 100% at baseline, 35.72% (95% CI: 0.22–71.21%) at 12 months, and 43% (95% CI: 0.00–91.52%) at 25 months. Significant differences were found between baseline and 12 months (*P* = 0.007), baseline and 25 months (*P* = 0.021) but not between 12 months and 25 months (*P* = 1.00). Both knockdown and mortality were higher at all ages for PermaNet 3.0 than PermaNet 2.0 (Fig. 2, Fig. 3).

Knockdown for Olyset Plus was 76.5% (95% CI: 34.95–100%) for baseline, 17% (95% CI: 0.00–53.81%) for 12 months and 12% (95% CI: 0.00–43.85%) for 25 months (Fig. 2). Differences between baseline and 12 months (*P* < 0.001) and baseline and 25 months (*P* < 0.0001) were significant but not between 12 and 25 months (*P* = 0.38). A similar reduction was observed for 24-h mortality; 56.96% (95% CI: 0.00–92.65%) for baseline, 10.09% (95% CI: 0.00–27.84%) for 12 months, and 8.70% (95% CI: 0.00–21.51%) for 25 months (Fig. 3). As with knockdown, there was a significant difference between baseline and 12 months (*P* < 0.001), baseline and 25 months (*P* < 0.001) but not between 12 and 25 months (*P* = 0.93). Both knockdown and mortality were higher at baseline for Olyset Plus than Olyset, but not at 12 or 25 months (Fig. 2, Fig. 3).

For the PBO nets, PermaNet 3.0 and Olyset Plus, knockdown and mortality were positively correlated (Spearman rank correlation, *ρ* = 0.76, *P* < 0.001, *n* = 150 and *ρ* = 0.66, *P* < 0.001, *n* = 120, respectively). For the pyrethroid-only nets, PermaNet 2.0 and Olyset, correlations were low and non-significant (*ρ* = 0.19, *P* = 0.018, *n* = 150 and *ρ* = 0.23, *P* = 0.013, *n* = 100, respectively).

#### 3.1.2 Impact of host presence

The results of the tests with a human host were compared to those without; the latter from Mechan et al. (2022) (Fig. 2, Fig. 3). No consistent differences were found between the cone assays with a host and without. There was no difference at baseline for PermaNet 3.0 with 100% effectiveness for both knockdown and mortality with and without a host (Fig. 2). For Olyset Plus, the addition of a host increased the mortality at baseline from 12.31% (95% CI: 1.80–22.82%) without a host to 44% (95% CI: 31.71–56.29%) with a host (Fig. 3). After baseline, neither PBO nor non-PBO nets showed significant differences in knockdown or mortality in comparisons between tests with and without a host (Fig. 2, Fig. 3).

### 3.2 Movement events

#### 3.2.1 Total movement events

**Fig. 4:**
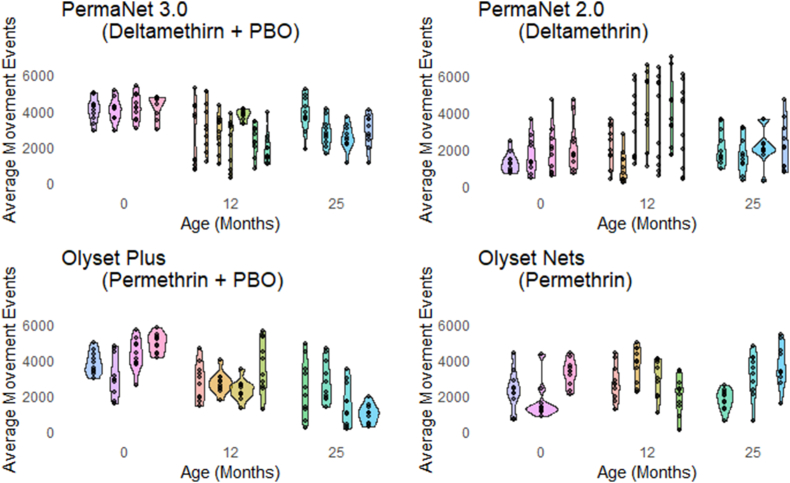
Violin plots displaying the number of movement events detected for all four net types at the three age points. The different colours represent different net samples, and each dot shows the movement for each repetition.

The number of mosquito movement events on PermaNet 3.0 nets decreased as the nets aged (Fig. 4). On baseline nets, 4139.5 (95% CI: 3934.6–4344.4) movement events were detected, reducing to 2683.3 (95% CI: 2387.6–2978.9) at 12 months and 3000.2 (95% CI: 2699.3–3301.2) at 25 months. Results for baseline and 12 months were significantly different (*P* < 0.001), as were baseline and 25 months (*P* < 0.001). There was no significant difference between 12 and 25 months (*P* = 0.354). Movement on PermaNet 2.0 remained consistent across different ages (Fig. 4).

Similar to PermaNet 3.0, mosquito movement on Olyset Plus nets was highest at baseline then reduced as the nets increased in post-deployment age (Fig. 4) from 4030.35 (95% CI: 3703.07–4357.63) in the baseline nets to 2861.13 (95% CI: 2523.35–3198.9) at 12 months and 1933.48 (95% CI: 1516.65–2350.3) at 25 months. Results were significantly different for baseline and 12 months (*P* = 0.001), baseline and 25 months (*P* < 0.001) and 12 and 25 months (*P* = 0.002). Movement on Olyset nets remained consistent across different net ages (Fig. 4).

Also shown in Fig. 4 is the amount of variation in movement detected between net samples that are the same type and age, this is particularly clear for PermaNet 3.0 baseline nets recording similar movement; however, 12 months and 25 months show variation, perhaps as a result of variation in use of washing patterns or use.

#### 3.2.2 Movement events over time

**Fig. 5:**
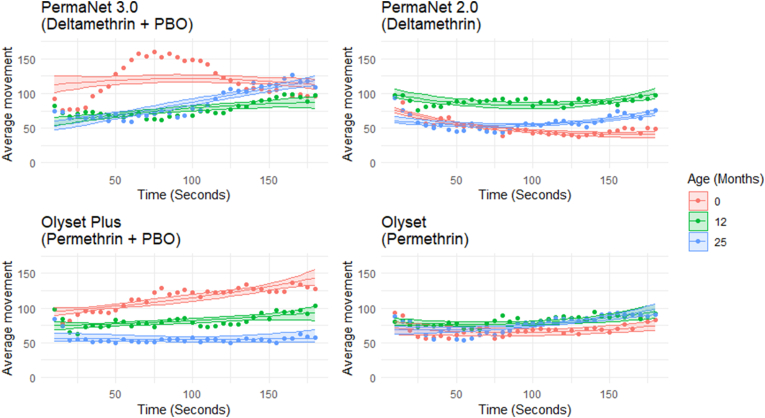
Scatter plots displaying the average movement detected over time for all four net types and three ages. Shaded areas indicate 95% CI as determined by the best-fit model. Each dot represents the mean of all observations at that time point.

Over the course of the exposure time, the mosquito movement on the different net types varied (Fig. 5). A general trend of increased movement with time was detected as shown in Fig. 5. PermaNet 3.0 baseline nets (Fig. 5) had the most mosquito movement in the second minute, mosquito movement on baseline nets were significantly different from 12 months (*P* < 0.001) and 25 months (*P* = 0.010). Mosquito movement on 12 months and 25 months nets were also significantly different (*P* < 0.001).

The movement of mosquitoes on PermaNet2.0 (Fig. 5) nets remained consistent across the 3 minutes for all three aged nets; however, mosquito movement on each aged net was significantly different (*P* < 0.001 for baseline and 12 months; *P* = 0.002 for baseline and 25 months; *P* < 0.001 for 12 months and 25 months).

Over the 3-min exposure mosquito movement on Olyset Plus nets (Fig. 5) aged 12 months and 25 months remained consistent, baseline nets showed a gradual increase of mosquito movement over time. Movement on each age group was significantly different (*P* < 0.001).

Olyset nets (Fig. 5) had similar mosquito movements detected across the 3-min exposure time. Baseline movement was significantly different from 12 months (*P* < 0.001), and 25 months (*P* = 0.027); however, no significant difference was observed between movement on 12 months and 25 months aged nets (*P* = 0.172).

#### 3.2.3 Relationship between movement with knockdown and mortality

**Fig. 6:**
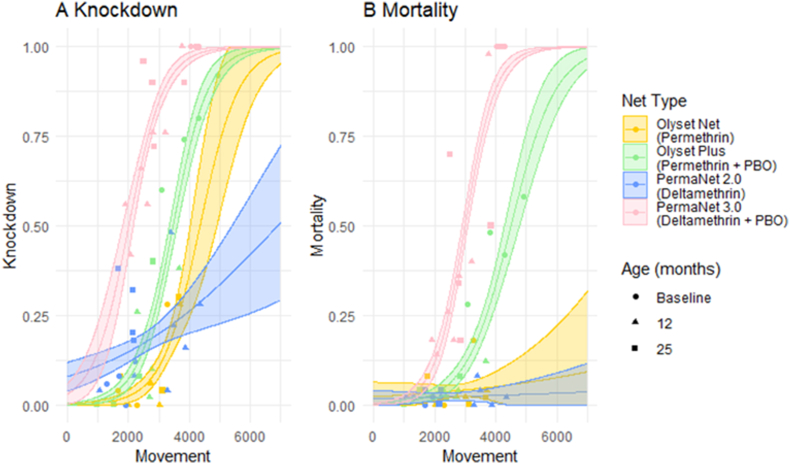
Scatter plots comparing the movement on all four net types at all three post-deployment ages with knockdown (**A**) and mortality (**B**). Shaded areas indicate confidence intervals as determined by the best-fit model.

There was a general pattern of increase in knockdown (Fig. 6A) and mortality (Fig. 6B) with increased mosquito movement events on the nets, but the strength of the effect varied between nets. Knockdown (Fig. 6A) increased more quickly as movement increased on PermaNet 3.0 compared to PermaNet 2.0 (*P* < 0.001). Olyset Plus and Olyset net also showed increased knockdown as movement increases; however, the increase for Olyset plus was stronger (*P* < 0.001).

For mortality (Fig. 6B), a clear relationship was shown between movement and mortality for PBO nets but for non-PBO nets increased movement was not associated with increased mortality (PermaNet 3.0: *P* < 0.001; Olyset Plus: *P* = 0.001)

### 3.3 Net contact

#### 3.3.1 Net contact over exposure time

**Fig. 7:**
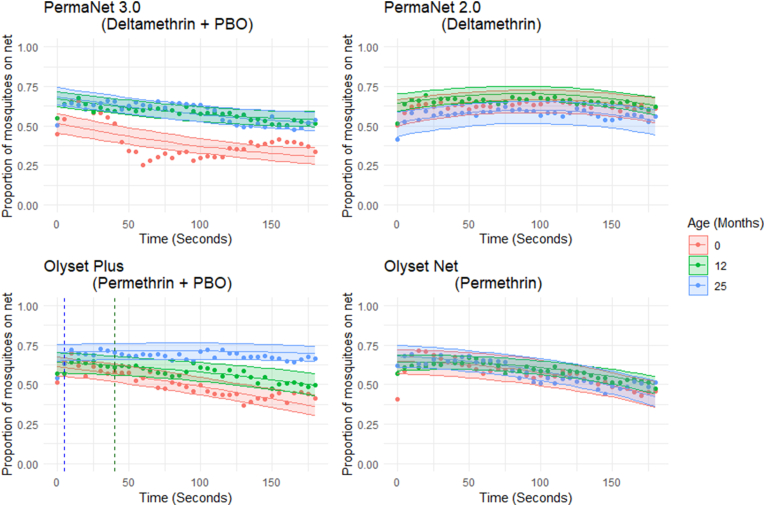
Scatter plots displaying the average number of mosquitoes on the net during the 3-min exposure time for all four net types. Shaded areas indicate 95% CI as determined by the best-fit model. Each dot represents the mean of all observations at that time point. The blue line on plot Olyset Plus indicates the point where 25 months becomes significantly different from baseline and the green line indicates where 12 months becomes significantly different from 25 months as calculated with *post-hoc* analysis.

Net contact is expressed as the proportion of mosquitoes exposed that are contacting the net within each time point (in 5-s intervals). Net contact was measured throughout the test for all four net types using scan sampling; results are displayed for each net type in Fig. 7.

Net contact time for PermaNet 3.0 was lowest at baseline (Fig. 7), with increases seen at later ages. Baseline net contact was significantly different from both 12 months and 25 months (*P* < 0.001 in both instances), but not between 12 and 25 months (*P* = 1.00). The best-fit model indicated that the contact time decreased across the time period of the exposure (*P* < 0.001): at the start of the test on baseline nets, there were an average of 2.25 mosquitoes in contact with the net and 1.68 at the end.

For PermaNet 2.0 nets, the amount of net contact was similar between the three ages (Fig. 7) and showed no significant change with time of exposure (*P* = 0.398).

As Olyset Plus nets increased in age, net contact generally increased (Fig. 7). The difference between baseline and 12 months was not significant (*P* = 0.11), but differences were significant between baseline and 25 months (*P* < 0.001) and 12 and 25 months (*P* = 0.003). *Post-hoc* analysis indicated no difference between baseline and 25 months until 5 s (blue line on Fig. 7) and until 40 s between 12 and 25 months (green line on Fig. 7). At the start of the test on baseline nets there were an average of 2.58 mosquitoes in contact with the net and 2.05 mosquitoes after exposure (*P* < 0.001).

In Olyset Nets the amount of net contact was similar between the three ages (Fig. 7), but there was a significant decline in the amount of contact over time of exposure (*P* < 0.001).

#### 3.3.2 Relationship between net contact with knockdown and mortality

**Fig. 8:**
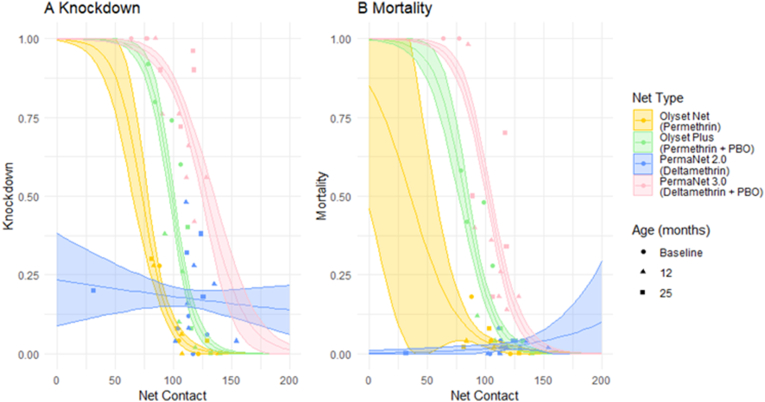
Scatter plots comparing the net contact on all four net types at all three post-deployment ages with knockdown (**A**) and mortality (**B**). Shaded areas indicate confidence intervals as determined by the best-fit model.

The relationship between net contact with knockdown and mortality is shown in Fig. 8. An overall trend of increase in net contact resulting in reduced knockdown was found (Fig. 8A), which links to previous results for movement (Fig. 6A) where more movement resulted in more knockdown, all of which were significantly different (*P* < 0.001 for all net combinations). Furthermore, the same trend was found for mortality (Fig. 8B) for all net types apart from PermaNet 2.0. Significant differences were found between all net types (*P* < 0.001); however, Olyset Net and PermaNet 2.0 showed no significant difference (*P* = 0.698).

## 4 Discussion

In this study we demonstrated that pyrethroid-resistant *An. gambiae* mosquitoes made less overall total contact with pyrethroid-PBO ITNs than nets treated with pyrethroid alone. Furthermore, during three-minute cone exposures, fewer mosquitoes were in contact with the pyrethroid-PBO ITNs at any one moment. Finally, mosquitoes exhibited much greater total movement activity when exposed to pyrethroid-PBO ITNs than pyrethroid-only ITNs. However, these effects were only observed with newly distributed nets, with 12- and 25-month-old nets showing neither reduced contact nor greater mosquito activity compared to a pyrethroid-only net. These findings indicate that (after a few seconds of initial contact), pyrethroid-PBO nets produce more irritancy behaviour than pyrethroid nets; however, this effect declines quickly with operational use. Given that pyrethroid-only nets still incited a substantial degree of movement activity, these findings indicate that even nets that show little bio-efficacy can still have an irritant effect.

Currently, the WHO recommends tunnel tests for measuring feeding inhibition with ITNs (which may be seen as a proxy for repellency); however, many laboratories are unable to perform tunnel tests due to ethical concerns. The video cone tests provide a scalable tool for assessing mosquito behaviour close to ITNs without the need for animal hosts. The video cone test is neither a replacement nor an equivalent for the tunnel test; however, we demonstrate here that the video cone test provides insights into mosquito behaviour in proximity to an ITN.

With the exception of higher mortality with a host at baseline Olyset Plus, the differences observed between tests with and without a host were small and non-significant. Hughes et al. (2022) found distinct differences in the behaviour of mosquitoes on nets with and without a host. However, effects on longevity were counter-intuitive, with significantly longer survival with a host following exposure to PermaNet 2.0, though this difference did not persist in mosquitoes allowed to blood feed after the test. Whether this apparent difference between studies is linked to differing mortality endpoints, different resistance levels in the strains tested and/or differences in the attractiveness of the host is unclear. Mosquito behaviour to full sized nets with a host has been recorded using a back lit imaging system, the results from which found more movement in *An. gambiae* in the presence of a host (Angarita-Jaimes et al., 2016). Nevertheless, it seems that the hypothesis of greater mortality with a host, in general or for a specific net type, is not supported by current evidence.

Overall, the results for both PBO-pyrethroid ITNs (PermaNet 3.0 and Olyset Plus) showed similar trends; both elicited high movement, knockdown, and mortality that decreased as the nets increased in age. In contrast, PermaNet 2.0 and Olyset nets, which do not contain PBO, showed low movement, knockdown, and mortality. These data suggest that PBO induced behavioural changes in the mosquitoes such as more movement and reduced net contact due to a restoration of the effect of the lethal insecticide.

PermaNet 3.0 nets have PBO present, and the nets may have induced more irritation than PermaNet 2.0 in the mosquitoes as a result, though the deltamethrin concentration is also much higher on the roof of PermaNet 3.0. Yet a similar difference was seen between Olyset Plus and Olyset Nets, which have the same pyrethroid concentration, reinforcing the explanation that the differences are attributable primarily to PBO. For both net types, this effect was reduced as the nets increased in age, most likely due to the reduction in PBO present on the nets, as found with HPLC analysis by Mechan et al. (2022). An insecticidal effect is expected to be required to result in increased irritancy behaviour, which in this case would be attributable to the PBO reducing the effectiveness of the metabolic resistance in the Busia colony mosquitoes resulting in greater toxic effects on behaviour as well as knockdown and mortality.

In Hughes et al. (2022) the net contact was reduced when a host was present, but less so for susceptible strains of mosquitoes, suggesting that resistance status played a role in host-seeking behaviour. Jones et al. (2023) used the ViCTA analysis to compare mosquito behaviours on nets. They found mosquitoes exposed to insecticide-treated nets reduced the time spent crawling on the net surface when compared to untreated nets. Whilst the present study did not compare to untreated nets, the findings are similar as baseline nets that contained the most insecticide had the least amount of net contact but the highest amount of movement events especially in PermaNet 3.0 and Olyset Plus.

Non-contact repellency was not quantified in this study due to the small volume in the test arena of the WHO cone, but we did not observe a difference in contact irritancy between pyrethroids, with mosquitoes showing similar patterns of behaviour, assessed *via* the number of movement events, for both deltamethrin and permethrin. Investigation of repellency could be addressed by running larger scale experiments such as room-level tracking analysis with this much larger test arena allowing for a better evaluation of avoidance.

The results from this study provide important information regarding the time at which PBO takes to influence mosquitoes, with rapid impact evident. The contact time for mosquitoes on PBO nets decreased after the first minute of exposure, indicating that the mosquitoes are already feeling toxic effects of the insecticide and thus reduce contact with the net, this is especially true for PermaNet 3.0 nets observed here. For non-PBO nets, this irritation is not observed, with less or no change in contact over time, suggesting that the effect may result from PBO enhancing insecticide effect.

The two analysis methods used for this study both provided valuable information regarding the mosquitoes’ behaviour when in contact with a bednet. The scan sampling method, whilst it took longer to produce data helped to highlight a reduction in net contact over the exposure time and the ViCTA results showed the variation in movement between different net types. Combined together, these data helped determine a conclusion that PBO increases the irritancy behaviour caused by pyrethroids. Where high movement was detected in ViCTA results, lower net contact was counted with scan sampling showing the methods could be potentially used interchangeably.

The study had some limitations. As only one host was used throughout this experiment, the results may have been affected by the specific personʼs attractiveness to mosquitoes. To improve the generality of the study, more hosts should be used with the same nets to ensure that this is not a contributing factor to movement. Links made between irritation and mortality may be somewhat artificial because tests were conducted within a cone, which limits the capacity of mosquitoes to escape as they might in a real-world setting. Further testing in larger room scale or hut trials would be required to provide more conclusive evidence of this link. Furthermore, to improve the comparison between host and no host, videos and movement analysis of cone assays without a host on the same net samples could be completed. Negative controls (untreated nets) were used to check for mortality not due to ITN exposure however were not performed at sufficient scale to quantify movement behaviour.

## 5 Conclusion

Irritancy is closely linked to the killing effect. More movement led to higher mortality and knockdown rates. Interestingly, fewer net contacts were recorded, indicating that prolonged contact was not necessary for knockdown or mortality, particularly in the presence of PBO. The addition of a host did not increase knockdown and mortality when compared to without a host.

## Ethical approval

Ethical approval not needed as the host test subject was the experiment operator.

## Funding

This work was supported, in whole or in part, by the 10.13039/100000865Bill & Melinda Gates Foundation (Investment ID INV-017131). Under the grant conditions of the Foundation, a 10.13039/100026877Creative Commons Attribution 4.0 Generic License has already been assigned to the authorʼs accepted manuscript version that might arise from this submission.

## CRediT authorship contribution statement

**Emma Reid:** Conceptualization, Data curation, Formal analysis, Investigation, Methodology, Resources, Visualization, Writing – original draft, Writing – review & editing. **Frank Mechan:** Formal analysis, Resources, Visualization, Writing – review & editing. **Jeff Jones:** Methodology, Software, Writing – review & editing. **Amy Lynd:** Resources, Writing – review & editing. **Janet Hemingway:** Funding acquisition, Supervision, Writing – review & editing. **Philip McCall:** Methodology, Writing – review & editing. **David Weetman:** Conceptualization, Funding acquisition, Project administration, Resources, Supervision, Visualization, Writing – review & editing.

## Declaration of competing interests

The authors declare that they have no known competing financial interests or personal relationships that could have appeared to influence the work reported in this paper. Given their role as Co-Editor, David Weetman had no involvement in the peer review of this article and has no access to information regarding its peer review. Full responsibility for the editorial process for this article was delegated to Professor Aneta Kostadinova (Editor-in-Chief).

## Data Availability

The data collected and analysed during the study are included within the article and its supplementary file.
